# GZD824 overcomes FGFR1‐V561F/M mutant resistance in vitro and in vivo

**DOI:** 10.1002/cam4.4041

**Published:** 2021-06-10

**Authors:** Kaili Jiang, Xia Tang, Jing Guo, Rui He, Shingpan Chan, Xiaojuan Song, Zhengchao Tu, Yuting Wang, Xiaomei Ren, Ke Ding, Zhang Zhang

**Affiliations:** ^1^ International Cooperative Laboratory of Traditional Chinese Medicine Modernization and Innovative Drug Development of Chinese Ministry of Education (MOE Guangzhou City Key Laboratory of Precision Chemical Drug Development School of Pharmacy Jinan University Guangzhou China; ^2^ Guangzhou Institutes of Biomedicine and Health Chinese Academy of Sciences Guangzhou China

**Keywords:** FGFR1, GZD824, resistance, V561F

## Abstract

Abnormallyactivated FGFR1 has been validated as a therapeutic target for differentcancers. Although a variety of FGFR inhibitors have shown benefit in manyclinical patients with FGFR1 aberration, FGFR1 mutant resistance such as V561Mmutation, has been reported. To date however, no FGFR inhibitors have beenapproved to treat patients with FGFR mutant resistance. Herein, we report that GZD824, athird generation ABL inhibitor (Phase II, China), overcomes FGFR1‐V561F/M mutant resistance in vitro and in vivo. GZD824potently suppresses FGFR1/2/3 with an IC_50_ value of 4.14 ± 0.96, 2.77 ± 0.082, and 8.10 ± 0.15 nmol/L. It effectively overcomes FGFR1‐V561F/M and other mutantresistance in Ba/F3 stable cells (IC_50_:8.1–55.0 nM), and effectively inhibits the growth of Ba/F3‐FGFR1‐V561F/M mutantxenograft tumors in vivo (TGI=73.4%, 49.8% at20mg/kg, p.o, q2d). GZD824may be considered to be an effective drug to treat patients with FGFR1 abnormalactivation or mutant resistance in clinical trials.

## Graphical abstract

FGFR1‐V561F/M mutation is resistant to erdafitinib, pemigatinib, BGJ398, and TAS120 but sensitive to GZD824. GZD824 strongly suppresses FGFR1‐3 and effectively overcomes FGFR1‐V561F, V561M, and other mutant resistance in vitro (IC_50_:8.1–55.0 nM) and in vivo (TGI=73.4%, 49.8% at 20mg/kg, p.o, q2d). GZD824 as a type II FGFR1 inhibitor overcomes FGFR1‐V561F/M mutant resistance.

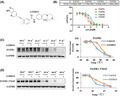

## 1 INTRODUCTION

The fibroblast growth factor receptor (FGFR) family consists of four isoforms FGFR1–FGFR4, whose natural ligands include a family of 22 fibroblast growth factors (FGF). The binding of FGFs and FGFRs induces receptor dimerization, recruits the fibroblast growth factor receptor substrate 2 (FRS2) adaptor proteins,^1^ then phosphorylates and activates the downstream signaling cascades *via* MAPK and PI3K/AKT.^2^ FGFR1 abnormalities have been found in a variety of cancers and play an important role in the growth, angiogenesis, migration, and resistance to cancer therapies.^3^ FGFR amplification has been frequently reported in lung squamous cell carcinoma (SqCC, 5.6%–24.8%), small cell lung cancers (SCLC, 5.6%–6%), estrogen receptor‐positive breast cancers (10%), and ovary (~5%) and bladder (<2%) cancers. FGFR1 fusions have been described in lung squamous cell carcinoma, breast cancer, glioblastoma, stem cell leukemia/lymphoma syndrome (SCLL), and myeloproliferative syndrome.^4^, ^5^ Less common activating mutations of FGFR1, such as N546K and K656E, have been identified in dysembryoplastic neuroepithelial tumor (DNET),^6^ small cell lung cancer (SCLC),^7^ Ewing sarcoma,^8^ and in other cancers.^9^

Two non‐selective FGFR1–3 inhibitors, erdafitinib (JNJ‐42756493) and pemigatinib (INCB054828) have been approved and are being marketed to treat bladder cancer and cholangiocarcinoma in patients with FGFR2 or FGFR3 rearrangements.^10^, ^11^ Several selective FGFR1–3 inhibitors, including infigratinib (NVP‐BGJ398),^12^ erdafitinib (JNJ42756493),^13^ AZD4547,^14^ and ICP‐192, have been developed and are being studied in different phase clinical trials. Other multiple kinase inhibitors, including ponatinib,^15^ dovitinib,^16^ lucitanib (E‐3810),^17^ and nintedanib (BIBF 1120),^18^ also have been shown to possess inhibitory activity against FGFRs. Currently, multiple clinical studies are in progress in patients with FGFR1 aberrantly activated cancers, including SCLL, breast, prostate, and lung cancer.^4^, ^11^

Although these FGFR inhibitors have shown benefits in many clinical patients with FGFR1 aberration, occurrence of FGFR1 mutant resistance has been reported. FGFR1 gatekeeper mutations such as V561M and N546K are the common mechanism of resistance to FGFR1–3 inhibitors such as BGJ398, AZD4547,^19^ lucitanib (E‐3810), and FIIN‐1 in cells or patients.^20^, ^21^ Some FGFRs inhibitors such as FIIN‐2,^22^ TAS‐120,^23^, ^24^ and LY2874455 ^25^ have been shown to overcome FGFRs mutant resistance but they are insensitive to FGFR2‐V564 M/F or FGFR3‐V555 M/F excluding LY2874455. In addition, it is unclear if FGFRs inhibitors overcome the FGFR1‐V561F mutation. There is, therefore, an unmet need to evaluate novel FGFR inhibitors which could overcome FGFR1‐V561F/M mutant resistance.

GZD824 (HQP1351, 3‐methyl‐4‐((5‐methyl‐7,8‐dihydropyrido[2,3‐d]pyridazin‐3‐yl)ethynyl)‐N‐(4‐((4‐methylpiperazin‐1‐yl)methyl)‐3‐(trifluoromethyl)phenyl)benzamide ^26^ is a third generation ABL inhibitor designed and synthesized by our group. It has completed Phase II clinical trials for chronic myeloid leukemia (CML) patients harboring Abl mutant resistant and is included in the priority review for new drug applications (NDA) by NMPA, China. Through a KINOMEscan screening of 442 kinases, we have reported that GZD824 possess binding activity with FGFR1–3, FLT3, and PDGFRα, but its activity against FGFR1 mutant resistance has not been disclosed.

Herein, we report the activities and mechanisms by which GZD824 can overcome FGFR1‐V561F/M mutant resistance in vitro and in vivo, and explore the potential applications of GZD824 in patients with FGFR aberrant activation and mutant resistance. We report that FGFR1‐V561F mutation is resistant to erdafitinib, pemigatinib, BGJ398, and TAS120 but is sensitive to GZD824. GZD824 strongly suppresses FGFR1–3 and effectively overcomes FGFR1‐V561F/M and other mutant resistance in vitro and in vivo, and may serve as a novel drug in clinical trials of cancer therapy.

## 2 MATERIALS AND METHODS

### 2.1 Agents

GZD824 was designed and synthesized by our laboratory. NVP‐BGJ398, TAS120, pemigatinib, and erdafitinib were purchased from the Selleckchem Company (Houston, TX, USA). These compounds were dissolved in DMSO (10 mM) and stored at −20℃. Primary antibodies against FRS2α (sc‐17841) were purchased from Santa Cruz Biotechnology (Santa Cruz, CA, USA). Primary antibodies against FGFR1(9740S), FGFR2(23328S), FGFR3(4574S), FGFR4(8562S), phosphor‐FGFR (3471S), phosphor‐FRS2α (3861S), FRS2α (sc‐17841), ERK1/2 (4695S), phosphor‐ERK1/2 (4370S), AKT(4685S), phosphor‐AKT (4060S), caspase‐3 (9662S), cleaved caspase‐3 (9664S), caspase‐9 (9502S), cleaved caspase‐9 (9505S), cyclin E1(20808S), cyclin D2 (3741S), cyclin B1 (12231S), CDK2 (2546S), CDK4 (12790S), GAPDH (2118), and secondary antibodies were purchased from Cell Signaling Technology (Boston, MA, USA).

### 2.2 In vitro kinase assays

FGFR1 and the Z′‐Lyte kinase assay kit were purchased from Invitrogen (Waltham, MA, USA) and the assays were performed according to the manufacturer's instructions as described previously.^27^

### 2.3 Cell cultures

FGFR sensitive cell lines, H1581, A204, KATOIII, NCI‐H716, SNU‐16, RT112, and MDA‐MB‐453 and other negative control cell lines MKN‐1, A549, and MDA‐MB‐231, were purchased from Shanghai Cell Bank (Type Culture Collection, China) or the American Type Culture Collection (ATCC). The base medium for MDA‐MB‐453 cell line is DMEM medium (CORNING, USA). All other cell lines were cultured in RPMI‐1640 medium supplemented with 10% FBS, 100 U/ml of penicillin, and 50 mg/ml of streptomycin in 5% CO_2_ at 37℃. The cells in the logarithmic growth phase were used for the experiments.

### 2.4 Construction of Ba/F3‐FGFR1 stable cells

The Ba/F3 cell lines stably expressing FGFR1, FGFR1‐V561M, FGFR1‐V561F, FGFR1‐K656N, and FGFR1‐K656E were self‐established through electroporation pCDNA3.1‐TEL plasmids using the Amaxa Cell Line Nucleofector Kit V (Lonza, Cologne, Germany). Stable cell lines were selected by G418 (Merck, USA) and then withdrawal of IL‐3 (R&D). All Ba/F3 stable cell lines were verified by positive drugs activities, DNA sequencing, and WB analysis. Parental Ba/F3 cells were maintained in RPMI 1640 supplemented with 10% FBS, 100 U/ml of penicillin, 50 mg/ml of streptomycin, and 10 ng/ml of IL‐3, while all Ba/F3 stable cell lines were cultured in the same medium without IL‐3.

### 2.5 In vitro cell proliferation Assay

Cells were seeded in 96‐well plate in complete medium (1000–6000 cells/well) and cultured for overnight. Then the cells were exposed to gradient concentrations (0.0005–10 μM) of compounds. Cell proliferation was analyzed by Cell Counting Kit 8 (CK04, Dojindo Laboratories, Japan) after co‐incubation for a further 72 h. IC_50_ values were calculated by fitting of concentration–response curves using GraphPad Prism 8.01 software. Each assay was repeated at least three times. Each IC_50_ value is displayed as mean ±SD of at least three independent experiments.

### 2.6 Cell cycle analysis

Cells were treated with indicated concentrations of GZD824 or DMSO for 24 h. About 6 × 10^5^ cells were re‐suspended in 150 μl of BD Cytofix/Cytoperm buffer solution I for 10 min at room temperature. The cell suspensions were added with BD Cytofix/Cytoperm buffer solution II and incubated with another 10 min at room temperature, and then incubated with 200 μl of propidium iodide buffer (0.1 mg/ml) in the dark for 10 min at 4℃. Assays were performed with a Guava easyCyte flow cytometer (Merck, USA).

### 2.7 Apoptosis assay

Cells were treated with indicated concentrations of GZD824 or DMSO for 48 h at 37℃. After incubation, cells were collected and washed twice with pre‐cold PBS. About 6 × 10^5^ cells were resuspended in 100 μl of 1× BD binding buffer solution (#556454, BD), and then stained with 7‐ADD (#559925, BD) and Annexin V‐PE (#556422, BD) in the dark for 15 min. Finally, 400 μl of 1×BD binding buffer solution were added to stop dyeing. The cells were then measured on a Guava easyCyte flow cytometer (Merck, USA).

### 2.8 Western blot analysis (WB)

Cells were treated with different concentrations of compound or DMSO for indicated time. Then, cells were lysed with 1 × SDS sample lysis buffer (recommended by CST). After ultra‐sonication and boiling, the cell lysates were loaded and then electrophoresed to 10%–12% SDS‐PAGE gel, and then the separated proteins were transferred to PVDF membranes. After blocking with 5% BSA in TBST solution (0.5% Tween‐20) at room temperature for 4 hrs, the membranes were incubated with the corresponding primary antibody (1:2000–1:200) at 4℃ overnight. After washing with TBST for 3–5 times, the HRP‐conjugated secondary antibodies were incubated for 2 hrs. The protein signals were detected with Amersham Imager 600 system (GE, Boston, MA, USA) using an ECL western blotting detection kit (Thermo Fisher Scientific, Waltham, MA, USA).

### 2.9 Induced fit docking studies

Ligands were built and prepared using LigPrep (Maestro Version 11.9, Schrodinger LLC, New York, NY, USA) with the default settings and the OPLS3e force field. The crystal structure of FGFR1 in complex with ponatinib (PDB code: 4V04) was extracted from the Protein Data Bank (http://www.pdb.org). The protein was prepared using the Protein Preparation Wizard workflow in Maestro to assign bond orders and add hydrogens. Crystal water molecules and all heteroatom (het) residues except those in ponatinib were removed. Docking studies were performed using the induced fit docking (IFD) protocol of Schrödinger (Schrödinger LLC, New York, NY, USA) software. The centroid of the cocrystal structure ligand ponatinib was used as the center of the grid box. Residues within 5 Å of the ligand poses were refined. The default protocols were used to perform induced fit docking.

### 2.10 Mouse xenograft tumor models

All animal studies were approved by the Institutional Animal Care and Use Committee. Male CB17‐SCID mice were purchased from Vital River Laboratory Animal Technology Inc. (Beijing, China). The right flanks of SCID mice were subcutaneously injected with 2 x 10^6^ Ba/F3‐FGFR1, Ba/F3‐FGFR1‐V561F, or Ba/F3‐FGFR1‐V561M cells, respectively. When the average tumor volume reached 100–200 mm^3^, mice bearing tumor were randomly divided into groups and treated with compounds or vehicle. All the compounds were suspended in 0.5% CMC‐Na aqueous solution. GZD824 (10 or 20 mg / kg, q2d), BGJ398 (qd), or vehicle (q2d) were administrated by gavage for 12–20 days. The body weight and tumor volume of animals were measured once every 2 days. Tumor volumes were calculated as L×W×W/2, where L and W are the length and width of the tumor, respectively. At the experiment terminal, tumor tissues were dissected, paraffin embedded, and sectioned for hematoxylin and eosin (H&E), immunohistochemistry (IHC), and TUNEL analysis.

### 2.11 Cellular thermal shift assay (CETSA)

Engagement between FGFR1 protein and GZD824 was assessed by CETSA. For each group, 2 × 10^7^ cells were collected and resuspended in PBS after treatment with GZD824 (1 μM for H1581, 100 nM for Ba/F3‐FGFR1‐V561F) or DMSO 4 h, then the cell suspensions of each group were divided into eight equal parts. Pairs consisting of one experimental aliquot and one DMSO aliquot were heated from 38.0 to 57.8℃ for 3 min. Then, the samples were exposed to liquid nitrogen for 10 min, and then placed in a water bath at 37℃ for 10 min. After three freeze–thaw cycles, cell lysates were centrifuged at 12000 rpm at 4℃ for 10 min, and then boiled for 15 min. The soluble supernatant was used for WB analysis.

### 2.12 Statistical analysis

Data are expressed as the mean ±SD of three independent experiments. Two‐tailed Student's *t*‐test was used for comparison between the two groups, and one‐way ANOVA was used for comparison among the multiple groups. Differences with *p* < 0.01 and *p* < 0.05 were considered very significant or significant, and marked as ** and *.

## 3 RESULTS

### 3.1 GZD824 strongly inhibits FGFR1–3 activities in vitro

**FIGURE 1:**
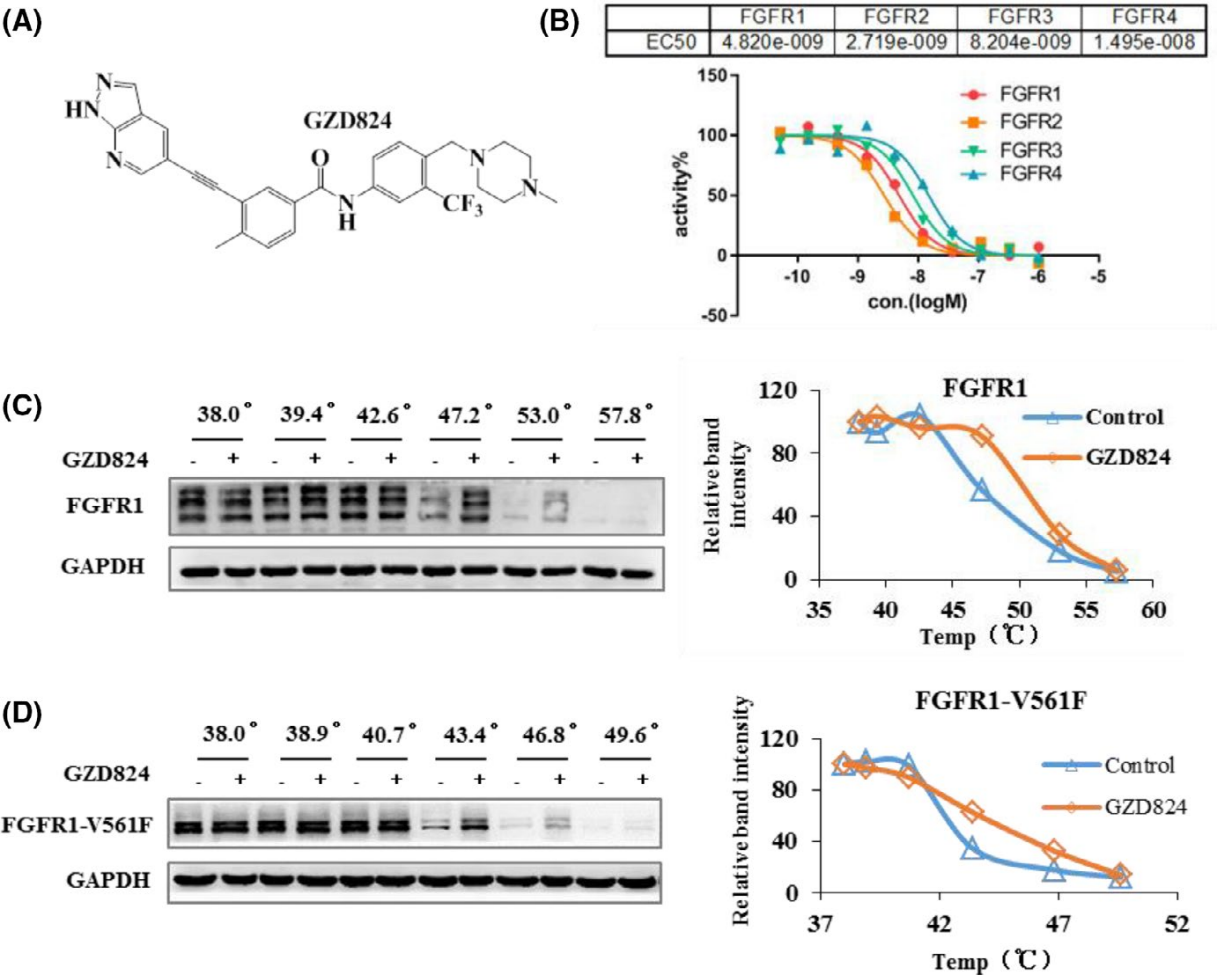
GZD824 is a pan‐FGFR inhibitor. (A) Chemical structure of GZD824; (B) GZD824 inhibits FGFR1/2/3/4 kinase activities in vitro by FRET‐based Z′‐Lyte assay; (C) GZD824 possess binding activities with FGFR1 in H1581 lung cancer cells by CETSA; (D) GZD824 possess binding activities with FGFR1‐V561F in Ba/F3‐FGFR1‐V561F cells by CETSA. Cells were treated with DMSO or GZD824 (1000 nM for H1581, 100 nM for Ba/F3‐FGFR1‐V561F) for 3 h, respectively. Drug‐treated cells were heated at gradient temperature from 38℃ to 57.8℃ for 3 min and then lysed, centrifuged to separate the soluble fractions from precipitates. The soluble fractions were analyzed by immunoblotting. Relative band intensities were plotted as a function of temperature followed a sigmoidal trend. The corresponding sigmoidal curve is on the right. GZD824 protected FGFR1 against thermal degradation, suggesting that the compound interacts with the receptor

We have developed GZD824 (Figure 1A) as an ABL inhibitor, and has also displayed its potential off‐target kinases to be FGFR1/2/3/4. To further explore the clinical applications of GZD824, the kinase inhibitory activities against FGFRs of GZD824 were evaluated using the FRET‐based Z′‐Lyte assay. Under the experimental conditions, GZD824 effectively inhibits FGFR1/2/3/4 with IC_50_ values of 4.14 ± 0.96, 2.77 ± 0.082, 8.10 ± 0.15, and 30.27 ± 21.66 nM, respectively (Table 1).

**TABLE 1:** IC_50_ and Kd values of GZD824 against FGFR1–4

FGFRs	Kd (nM)	IC_50_ (nM)
FGFR1	7.3 ± 0.3	4.14 ± 0.96
FGFR2	15.5 ± 3.5	2.77 ± 0.082
FGFR3	17.0 ± 1.4	8.10 ± 0.15
FGFR4	19.0 ± 1.4	30.27 ± 21.66

Binding activities were performed by KINOME*scan*™ assay. Kinase inhibitory was performed by Z′‐Lyte method‐based FRET principle.

A commercial binding affinity assay, KINOME*scan*™ was used to evaluate the potential activities of GZD824 against FGFR mutant resistance. It was shown that GZD824 possesses high binding affinities with FGFR1/2/3/4, with K_d_ values of 7.3 ± 0.3, 15.5 ± 3.5, 17.0 ± 1.4, and 19.0 ± 1.4 nM (Table 1), respectively, and also displays strong binding affinities to mutants FGFR2‐N550K, FGFR3‐G697C, FGFR3‐K650E, and FGFR3‐V555 M, with K_d_ values of 17.0 ± 5.7, 39.5 ± 7.8, 11.0 ± 0.0, and 19.5 ± 4.9 nM, respectively. We also investigated the binding properties of GZD824 with FGFR1 by a CETSA. The results showed that in the presence of GZD824, the stability of FGFR1 or FGFR1‐V561F proteins, but not of the control protein GAPDH, was significantly enhanced (Figure 1C and 1D). These results show that GZD824 can inhibit the activation of FGFRs and overcome FGFR mutant resistance.

### 3.2 GZD824 suppresses the proliferation of FGFR1–3‐driven cancer cells in vitro

To broadly assess the antiproliferative activities of GZD824 against cancer cells with FGFRs abnormal activation, we selected more than 10 solid cancer cells, including H1581 and A204 (FGFR1 amplification), KATOIII, NCI‐H716, and SNU16 (FGFR2 amplification), RT112 (FGFR3‐TACC3), MDA‐MB‐453 (FGFR4‐Y367C mutant activation), and other FGFRs with low expression of cancer cells as negative controls. The expression and activation levels of FGFR1–4 in these cells were determined *via* western blot experiments (Figure S1).

The results revealed that GZD824 exerted strongly antiproliferative activity against the cancer cells harboring FGFR aberrations in vitro, especially in FGFR1–FGFR3 cell lines, with IC_50_ values of 4.0–91.0 nM (Table 2). However, the activity of GZD824 against A549 and MDA‐MB‐231 cancer cells was minor with IC_50_ values of >1.0 μM, since no FGFR expression was observed (Table 2). Under the experimental conditions, the IC_50_ value of GZD824 is similar to that of BGJ398, but lower than that of ponatinib.

**TABLE 2:** GZD824 inhibits proliferations in different cancer cells driven by FGFRs activation

μΜ	FGFRs status	GZD824	BGJ398	FGF401	Ponatinib
H1581	FGFR1 amplification	0.0108 ± 0.0008	0.0194 ± 0.0025	12.885 ± 4.046	0.1280 ± 0.0191
A204	FGFR1 amplification	0.0244 ± 0.0062	/	8.248 ± 0.708	0.1690 ± 0.1343
KATOIII	FGFR2 amplification	0.0098 ± 0.0012	0.0171 ± 0.0024	9.3827 ± 2.8634	0.0187 ± 0.0008
NCI‐H716	FGFR2 amplification	0.0040 ± 0.0005	0.0042 ± 0.0008	11.124 ± 1.668	0.0264 ± 0.0029
SNU16	FGFR2 amplification	0.0144 ± 0.0048	0.0186 ± 0.0063	/	0.0782 ± 0.0102
RT112	FGFR3‐TACC3	0.0910 ± 0.0057	0.2334 ± 0.0767	14.523 ± 2.363	0.2161 ± 0.0082
MDA‐MB−453	FGFR4‐Y367C	0.1482 ± 0.1044	2.6977 ± 0.1039	0.0623 ± 0.0277	0.7067 ± 0.0264
MDA‐MB−231	/	1.998 ± 0.7819	>10	>10	0.7011 ± 0.0699
A549	/	3.017 ± 0.1965	8.7333 ± 1.9281	>10	0.9896 ± 0.0541
MKN−1	/	0.7426 ± 0.0318	5.107 ± 0.481	>10	0.758 ± 0.072

The antiproliferative activities of the compounds were evaluated using CCK‐8 assay. The cells were treated with compound or 0.1% DMSO for 72 h. Each data is expressed as mean ±SD of at least three independent experiments.

### 3.3 GZD824 overcomes FGFR1‐V561F/M mutant resistance in vitro

On the basis of the K_d_ assay, we posited that GZD824 possesses the potential to overcome FGFR1 gatekeeper mutant resistance. There is, however, no commercially available FGFR1 mutant kinase to validate this. Therefore, we constructed Ba/F3 model cells stably expressing FGFR1‐WT, K656E, K656N, V561M, and V561F mutations. We evaluated the antiproliferative activities of GZD824, two approved FGFRs inhibitors (pemigatinib and erdafitinib), a selective FGFR1/2/3 inhibitor (BGJ398), an irreversible pan‐FGFR1–4 inhibitor (TAS120), and multiple kinase ponatinib inhibitors in Ba/F3 stable cells. It was shown that the two marketed FGFR inhibitors were insensitive to these four FGFR1 mutants, with a 9.3‐ to 218‐fold increase in the IC_50_ values. BGJ398 also displayed resistance to V561F, V561M, and K656E with IC_50_ values of >330 nM, and excluded FGFR1‐K656N mutation (IC_50_ = 0.082 ± 0.0055 μΜ). The same as the reported FGFR1‐V561M kinase inhibitory activities (18.6 vs. 1.6: V561M vs. WT), ponatinib displayed a 10‐fold increase of IC_50_ value (0.40 ± 0.011 μΜ vs. 0.033 ± 0.0028 μΜ) in Ba/F3‐FGFR1‐V561M compared to Ba/F3‐FGFR1‐WT cells. We also found that TAS120 was insensitive to FGFR1‐V565M and FGFR1‐V561F, with IC_50_ values of 1.46 ± 0.12 μΜ and 1.00 ± 0.067μM, respectively, but it retains its sensitivity to K656E (0.017 ± 0.0022 μΜ) and K656N (0.010 ± 0.0022 μΜ) (Table 3).

**TABLE 3:** GZD824 overcomes FGFR1 mutant resistance in Ba/F3 models

IC_50_ (μΜ)	GZD824	Pemigatinib	Erdafitinib	BGJ398	TAS120	Ponatinib
Ba/F3‐FGFR1	0.0081 ± 0.0011	0.015 ± 0.0015	0.023 ± 0.003	0.031 ± 0.0029	0.0068 ± 0.0006	0.033 ± 0.0028
Ba/F3‐FGFR1‐V561M	0.055 ± 0.0037	2.31 ± 0.35	2.59 ± 0.26	4.38 ± 0.28	1.46 ± 0.12	0.40 ± 0.011
Ba/F3‐FGFR1‐V561F	0.012 ± 0.0005	3.28 ± 0.10	2.49 ± 0.56	4.25 ± 0.064	1.00 ± 0.067	0.056 ± 0.0090
Ba/F3‐FGFR1‐K656E	0.0206 ± 0.0008	0.14 ± 0.0097	0.24 ± 0.048	0.33 ± 0.0029	0.017 ± 0.0022	0.088 ± 0.028
Ba/F3‐FGFR1‐K656N	0.012 ± 0.0020	0.025 ± 0.0063	0.13 ± 0.0088	0.082 ± 0.0055	0.010 ± 0.0022	0.047 ± 0.0088
Ba/F3(+IL‐3)	1.71 ± 0.59	8.98 ± 1.40	3.40 ± 0.032	5.66 ± 0.88	>10	2.38 ± 0.42

The antiproliferative activities of the compounds were evaluated using CCK‐8 assay. The cells were treated with compound or 0.1% DMSO for 72 h. Each data is expressed as mean ±SD of at least three independent experiments.

We found that GZD824 can strongly inhibit FGFR1‐V561M, FGFR1‐V561F, and other K656E, K656N mutants in Ba/F3 models, with IC_50_ values of 0.055 ± 0.0037, 0.012 ± 0.0005, and 0.0206 ± 0.0008 and 0.012 ± 0.0020 μM, respectively (Table 3). This implies that GZD824 has a strong potential to overcome FGFR1 mutant resistance.

### 3.4 GZD824 inhibits phosphorylation of FGFR1 and downstream proteins

**FIGURE 2:**
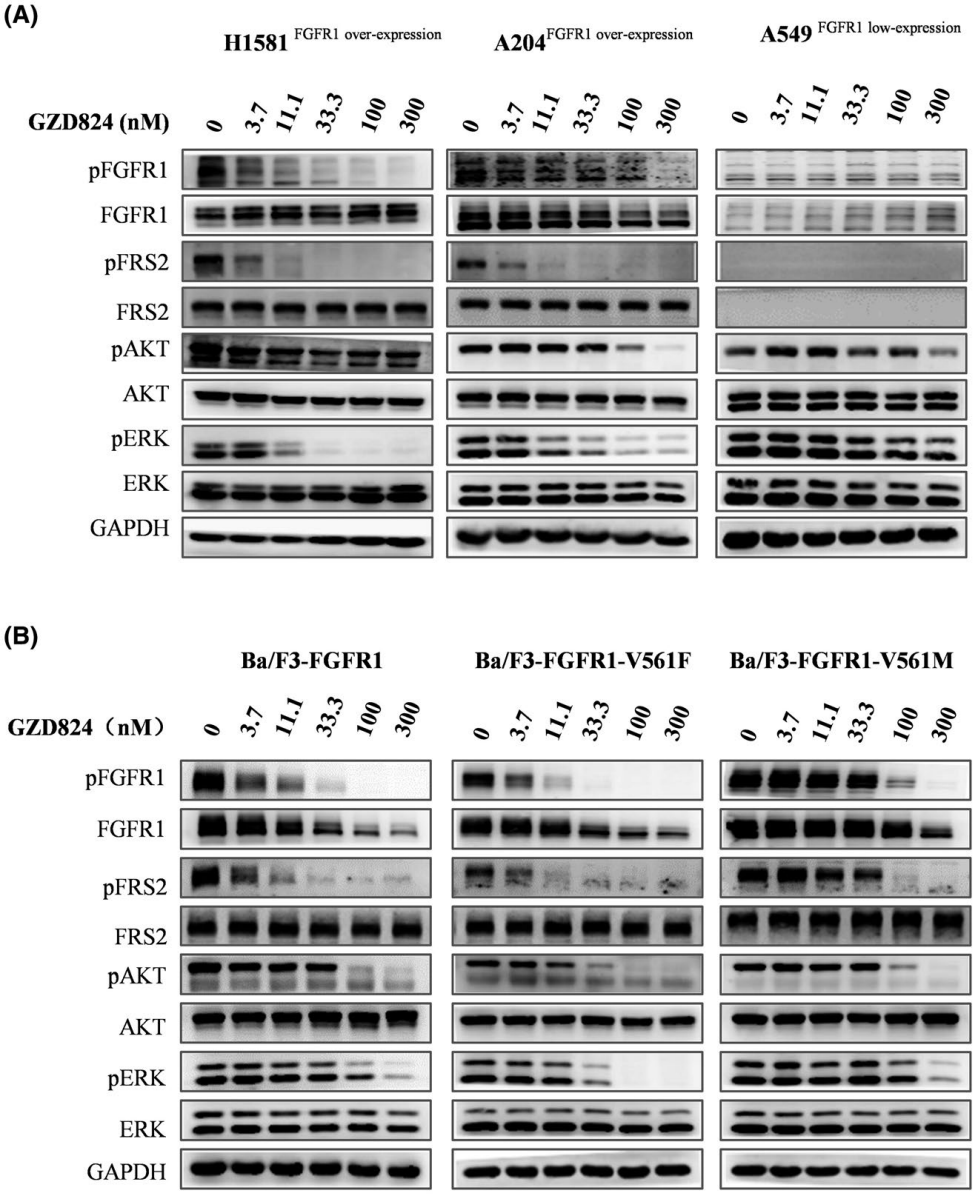
GZD824 suppresses FGFR1‐WT and V561F/M mutant signal pathway in cells (4 h). (A) Effects of GZD824 on FGFR1 signal pathway in H1581, A204, and A549 cancer cell lines. (B) Effects of GZD824 on FGFR1 signal pathway in Ba/F3 model cells. Cells were treated with or without compound GZD824 for 4 h at the indicated concentration, then harvested and lysed. Cell lysates were separated by sodium dodecyl sulfate‐polyacrylamide gel electrophoresis (SDS‐PAGE) and analyzed by western blot for phosphorylated FGFR1, FGFR1, phosphorylated FRS2, FRS2, phosphorylated AKT (Ser473), AKT, phosphorylated Erk, and Erk. GAPDH was used as a control

To study the mechanism of action of GZD824, the effects of GZD824 on the FGFR1 signaling pathway were investigated in H1581^FGFR1 overexpression^, A204 ^FGFR1 overexpression^, Ba/F3‐FGFR1, Ba/F3‐FGFR1‐V561F, Ba/F3‐FGFR1‐V561M, and A549 ^FGFR1 lowexpression^ by western blotting analysis. As shown in Figure 2, treatment with GZD824 for 4 h revealed dose‐dependent inhibition of FGFR1 phosphorylation, the downstream FRS2 and ERK1/2 proteins progressing from 3.7 nM to 300 nM in H1581, Ba/F3‐FGFR1, and Ba/F3‐FGFR1‐V561F cells.

GZD824 displayed no obvious effect on the total level of FGFR1 protein expression in H1581, A204, and A549 cell lines (Figure 2A) but showed an obvious degradation of FGFR1 in all Ba/F3 stable cells at doses of 33.3 nM or higher, with an increased ubiquitination level of FGFR1 (Figure S2B). Further research validated that the proteasome inhibitor MG132 can rescue FGFR1 degradation induced by GZD824, which supports that this is a proteasome‐dependent pathway (Figure S2A). However, its effects on FGFR1 downstream signal pathway in A549 cells were very weak as a negative control.

Under the same conditions, upon treatment with 300 nM of pemigatinib, TAS120 or BGJ398 in Ba/F3‐FGFR1‐V561F and Ba/F3‐FGFR1‐V561M, no effects on activation of FGFR1 and its signal pathway were observed (Figure S3).

### 3.5 GZD824 induces G0/G1 phase arrest and apoptosis in Ba/F3‐FGFR1‐V561F cells

**FIGURE 3:**
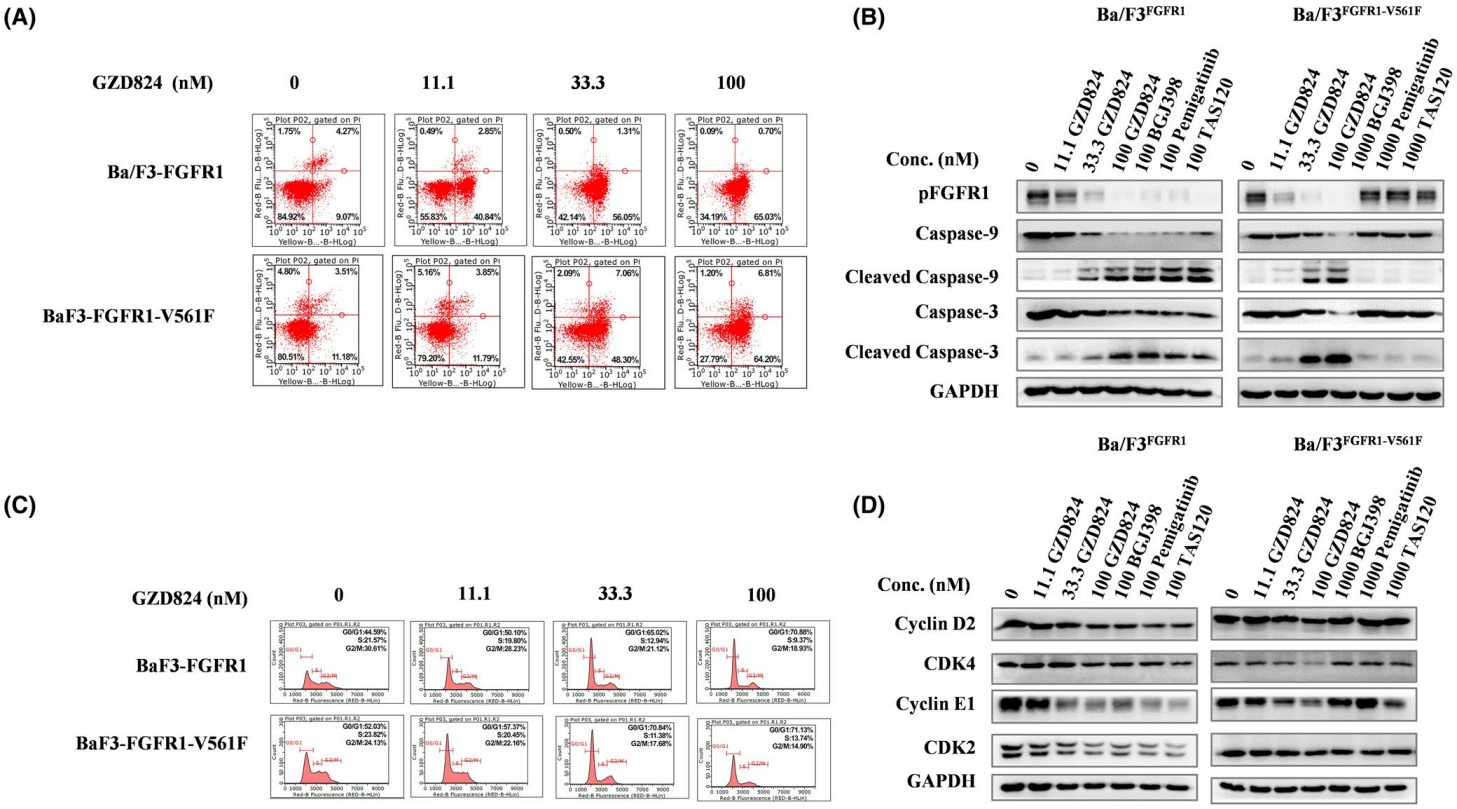
GZD824‐induced G0/G1 phase arrest and apoptosis in Ba/F3‐FGFR1‐WT and Ba/F3‐FGFR1‐V561F cells. (A) GZD824‐induced apoptosis in Ba/F3‐FGFR1 and Ba/F3‐FGFR1‐V561F cells. Cells were treated with various doses of GZD824 for 48 h before DNA labeling by Annexin V and 7‐AAD and cell apoptosis analysis by flow cytometry. Representative flow cytometry profiles were shown here. (B) Effects of GZD824 on apoptosis related proteins of capase‐3 and capase‐9 by western blotting analysis. (C) GZD824‐induced G0/G1 phase arrest in Ba/F3‐FGFR1 and Ba/F3‐FGFR1‐V561F cells. Cells were treated with various doses of GZD824 for 24 h before DNA labeling with propidium iodide and cell cycle analysis by flow cytometry. Representative flow cytometry profiles of cell cycle distribution were shown here. (D) Effects of GZD824 on cell cycle‐related proteins of Cyclin D2, CDK4, Cyclin E1, and CDK2 by western blotting analysis

To further identify the efficacy of GZD824, the effects of GZD824 on cell cycle and apoptosis in Ba/F3‐FGFR1 and Ba/F3‐FGFR1‐V561F cell lines were detected by flow cytometry analysis. The results displayed that GZD824 dose‐dependently induces G0/G1 phase arrest and apoptosis in Ba/F3‐FGFR1 and Ba/F3‐FGFR1‐V561F. Treatment with 100 nM of GZD824 led to 70.88% and 71.13% G0/G1 phase arrest after 24 h, and 65.73% and 71.01% apoptosis after 48 h in Ba/F3‐FGFR1 and Ba/F3‐FGFR1‐V561F cells, respectively (Figure 3A and 3C). Under the same conditions, even treatment with 1000 nM of NVP‐BGJ398, pemigatinib, and TAS120 showed a weak effect on cell cycle and apoptosis in Ba/F3‐FGFR1‐V561F cells (Figure S5). WB analysis also showed that GZD824 dose‐dependently decreases the protein levels of CDK2, cyclin D2, CDK4, cyclin E, and the activating cleavage of caspase‐3 and caspase‐9 in the above FGFR1 model cells (Figure 3B and 3D).

### 3.6 GZD824 as a type II FGFR1 inhibitor overcomes FGFR1‐V561F/M mutant resistance

**FIGURE 4:**
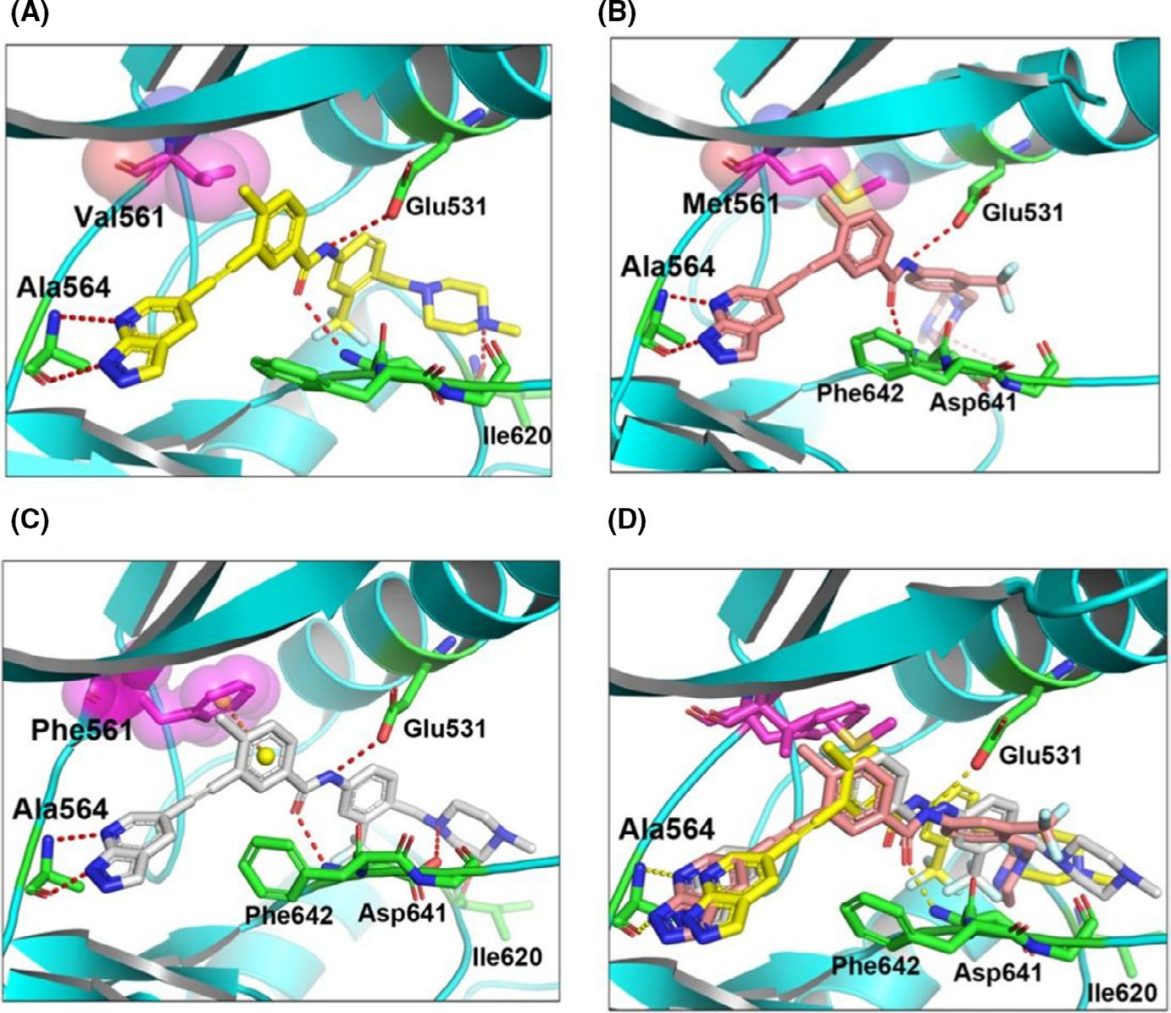
Binding mode analysis of GZD824 to FGFR1^WT^, FGFR1^V561M^, and FGFR1^V561F^. (A) Molecular docking of GZD824 into FGFR1^WT^ (generated from PDB code 4V04). (B) Molecular docking of GZD824 into FGFR1^V561M^ (generated from PDB code 4V04). (C) Molecular docking of GZD824 into FGFR1^V561F^ (generated from PDB code 4V04). (D) Overlay of the binding conformation of GZD824 from the FGFR1^WT^, FGFR1^V561M^, and FGFR1^V561F^, respectively

In order to explain the mechanism of sensitivity of GZD824 to the FGFR1‐V561M/F mutation, we performed an induced fit docking. The docking results of GZD824 with FGFR1 (PDB code: 4V04) shown in Figure 4 revealed that GZD824 as a type II inhibitor binds to the DFG‐out conformation of FGFR1‐WT, FGFR1‐V561M, and FGFR1‐V561F. The 1H‐pyrazolo[3,4‐b]pyridine core of GZD824 occupies the adenine pocket of FGFR1 kinase and forms hydrogen bond interactions with the backbone of Ala564 in the hinge region of the FGFR. The methylphenyl ring occupies the hydrophobic pocket located behind the gatekeeper residue Val561. The amide moiety is involved in two hydrogen bond interactions with the backbone nitrogen atom of Asp641 from the DFG motif and the side chain of the strictly conserved glutamate of the aC‐helix (Glu531), which is characteristic of type II inhibitors. The side chain of Phe642 is extended toward the solvent, creating an induced‐fit hydrophobic pocket into which the trifluoromethylphenyl ring binds. The terminal piperazinyl nitrogen atom of GZD824 is protonated and the resulting tertiary ammonium ion forms a hydrogen bond with the carbonyl oxygen atom of residue Ile620 in the catalytic loop. As shown in Figure 4, GZD824 binds to FGFR1‐V561M in a very similar fashion to that adopted by FGFR1‐WT. Although the 2.8 Å increase in length that occurs upon replacing a valine residue with methionine, GZD824 could participate in favorable van der Waals interactions with the gatekeeper Met without causing steric clashes. In the GZD824‐FGFR1 V561F docking complex, the side chain of Phe561 rotates to conserve the electrostatically favorable face‐to‐face π–π interaction with the methylphenyl ring of GZD824.

Ponatinib binds to FGFR1 WT, FGFR1 V561M, and FGFR1 V561F in a very similar fashion to GZD824. BGJ398, pemigatinib, and TAS120 are not type II inhibitors and reasonable binding conformations are not found between these and FGFR1‐V561M or FGFR1‐V561F.

### 3.7 GZD824 overcomes FGFR1‐V561F and V561M mutant resistance in vivo

**FIGURE 5:**
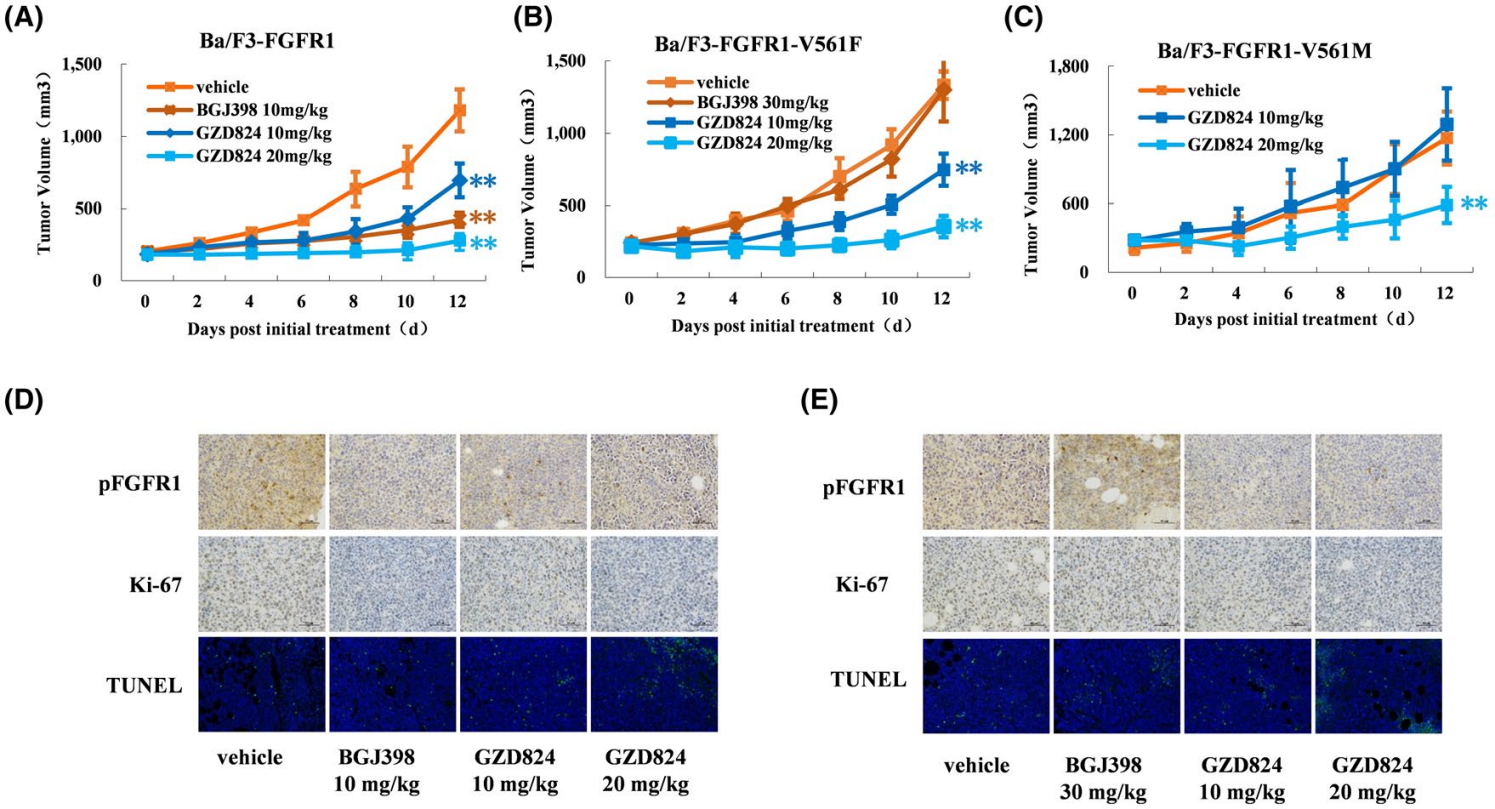
In vivo effect of GZD824 in Ba/F3‐FGFR1, Ba/F3‐FGFR1‐V561M, and Ba/F3‐FGFR1‐V561F xenograft model. (A) Antitumor efficacy of GZD824 in Ba/F3‐FGFR1 xenograft mouse model. (B) Antitumor efficacy of GZD824 in Ba/F3‐FGFR1‐V561F xenograft mouse model. (C) Antitumor efficacy of GZD824 in Ba/F3‐FGFR1‐V561M xenograft mouse model. (D) pFGFR1, Ki67, and TUNEL staining of Ba/F3‐FGFR1 tumors harvested from mice treated with GZD824, BGJ398, or vehicle for 12 days. (E) pFGFR1, Ki67, and TUNEL staining of Ba/F3‐FGFR1‐V561F tumors harvested from mice treated with GZD824, BGJ398, or vehicle for 12 days. Tumors harvested from the mice after dosing vehicle, GZD824, or BGJ398 for 12 days. CB17‐SCID mice xenograft models were orally dosed once every 2 days (q2d) for 12 days with vehicle or GZD824, or once a day (qd) with BGJ398. Tumor volumes were measured every 2 days. Tumors were harvested from the mice at the terminal. (*p < 0.05, **p < 0.01)

We further investigated the antitumor efficacy of GZD824 in Ba/F3‐FGFR1, Ba/F3‐FGFR1‐V561M, and Ba/F3‐FGFR1‐V561F mouse xenograft models. The animals were administered with vehicle, GZD824 once every 2 days at 10 mg/kg/day or 20 mg/kg/day or with BGJ398 once every day (10 mg/kg for Ba/F3‐FGFR1 and 30 mg/kg for Ba/FGFR1‐V561F) *via* oral gavage for 12 consecutive days. GZD824 displayed no mortality or significant loss of body weight observed during experiment (Figures S4A, S4B and S4C). GZD824 exhibited strong dose‐dependent antitumor efficacy with tumor growth inhibition (TGI) values of 76.5%, 49.8%, and 73.4% at dosages of 20 mg/kg (Bid) in Ba/F3‐FGFR1, Ba/F3‐FGFR1‐V561M, and Ba/F3‐FGFR1‐V561F, respectively (Figure 5A, 5B, and 5C). BGJ398 also displayed a TGI value of 64.2% in the Ba/F3‐FGFR1 xenograft model, but showed almost no effect on Ba/F3‐FGFR1‐V561F models even with a dose of 30 mg/kg (TGI=2.4%). IHC and TUNEL assays in the tumor tissues displayed that GZD824 inhibits the FGFR1 activation and induces apoptosis in Ba/F3‐FGFR1 and Ba/F3‐FGFR1‐V561F xenograft models (Figure 5D and 5E).

## 4 DISCUSSION

FGFR1–FGFR4 exhibit various abnormal activations in different tumors, including overexpression, mutation and fusion, drive malignant tumor proliferation, migration, and invasion. These observations suggest that FGFR1–FGFR4 may be potential targets of broad‐spectrum targeted therapy for many cancers. The clinical treatment of FGFR2 and FGFR3 abnormal tumors has been successful, and two marketed drugs (erdafitinib and pemigatinib) have been approved to treat those two kinds of tumors, respectively. Although dozens of FGFR inhibitors have been developed, they have not been used successfully in the treatment of patients with abnormal FGFR1/4 activation and are still at the stage of clinical research.

FGFR1 can be considered a therapeutic target for many cancers from different tissues.^28^ It has been reported that FGFR1 amplifications emerge in various of cancers, such as NSCLC (12%–17%), prostate cancer (16%), myxofibrosarcoma (20%), bladder cancer (9%), esophageal cancer (9%), colorectal cancer (6%), and in undifferentiated pleomorphic sarcomas (7%).^3^, ^4^ FGFR1 fusions such as BCR–FGFR1 ^29^ and FGFR1OP2‐FGFR1 ^30^ have been found in acute myeloid leukemia and other cancers. Due to resistance mediated by target mutation or bypass signal activation, treatment of FGFR1 amplified cancers with FGFR inhibitors is currently unsatisfactory. Reported data indicate that cancer cells harboring FGFR1 amplifications can acquire resistance to FGFR inhibitors mediated by overexpression of NRAS, MET amplification, mutational inactivation of PETN, and activation of AKT.^31^ Gatekeeper mutations such as FGFR1‐V561M (FGFR2‐V564F/I, FGFR3‐V555M) are, however, the predominant resistant mechanism during treatment with FGFR‐targeted therapies.^19^ Although many FGFR inhibitors such as erdafitinib, pemigatinib, and TAS120 have been developed, it has been reported that only LY2874455 and ponatinib can overcome FGFR1‐V561M mutant resistance.

The gatekeeper mutation could generate a steric clash, or eliminate critical hydrogen bonds required high‐affinity binding preventing inhibitors binding. Crystal structure of FGFR1‐V561M displayed that this gatekeeper mutation can stabilize the hydrophobic spine to promote catalytic activation, which was validated by FGFR1‐V561M conferring a 38‐fold increase in k_cat_ relative to FGFR1‐WT by a kinase kinetic assay.^32^ FIN‐2, FIN‐3, and TAS‐120, which have been designed to form a covalent bond with FGFR kinases to overcome FGFRs mutant resistance, were all successful in overcoming FGFR2‐K659N, C491A, and other mutations but not FGFR2‐V564F in cells or patients. In this paper, we first report the sensitives of FGFR1‐V561F to FGFR inhibitors which similar to V561M are insensitive to pemigatinib, erdafitinib, BGJ398, and TAS120. We were surprised to find that GZD824 can effectively overcome V561F mutant resistance in cells and animals with an IC_50_ lower than that of ponatinib. Our induced fit docking assays suggested that GZD824 possess a different binding model compared to those of type I inhibitors, which bind to FGFR1‐V561F/M in a very similar fashion to FGFR1‐WT, a type II FGFR1 inhibitor.

It has been reported that BGJ398 effectively inhibits the activation of ERK but not AKT in FGFR1‐amplified lung cancer cell lines.^1^ In this research, GZD824 was found to exert properties similar to those of BGJ398 in H1581 and A204 cells, which displays obvious inhibition of pFRS2 and pERK at a dose of 3.7 nM or higher, but with no effect on pAKT, even at a dose of 300 nM. In FGFR2 overexpressed SNU‐16 and in NCI‐H716 cells, GZD824, at levels from 3.7 to 300 nM can strongly inhibit pAKT. We suggest these differences may be due to the different backgrounds of the cell lines.

In summary, we report GZD824 as a pan‐FGFR inhibitor which inhibits the signaling pathways of FGFR1 kinases and suppresses the proliferation of cancer cells harboring overexpression or mutant activation of FGFR1 in vitro and in vivo. GZD824 overcomes FGFR1‐V561F, V561M, K656E, and K656N mutant resistance, and effectively inhibits the growth of Ba/F3‐FGFR1‐V561F/M mutant xenograft tumors in vivo. GZD824 may be considered to be an effective drug to treat patients with FGFR1 abnormal activation or mutant resistance in clinical trials.

## CONFLICT OF INTEREST

All authors declare that they have no conflict of interest.

## ETHICAL STATEMENT

All animal studies were approved by the Institutional Animal Care and Use Committee of the Medical College of Jinan University. This article does not contain any studies with human performed by any of the authors. Informed consent was obtained from all individual participants included in the study.

## Supporting information

Fig S1‐S5

## Data Availability

The data that support the findings of this study are available from the corresponding author upon reasonable request.
