# Supplementary material for: GZD824 overcomes FGFR1‐V561F/M mutant resistance in vitro and in vivo
Source: Cancer Med. 2021 Jun 10;10(14):4874–84. doi: 10.1002/cam4.4041 (PMC8290231; doi:10.1002/cam4.4041)
Supplement: Supplementary file 1 — Fig S1‐S5 [file CAM4-10-4874-s001.pptx]

## Slide 1
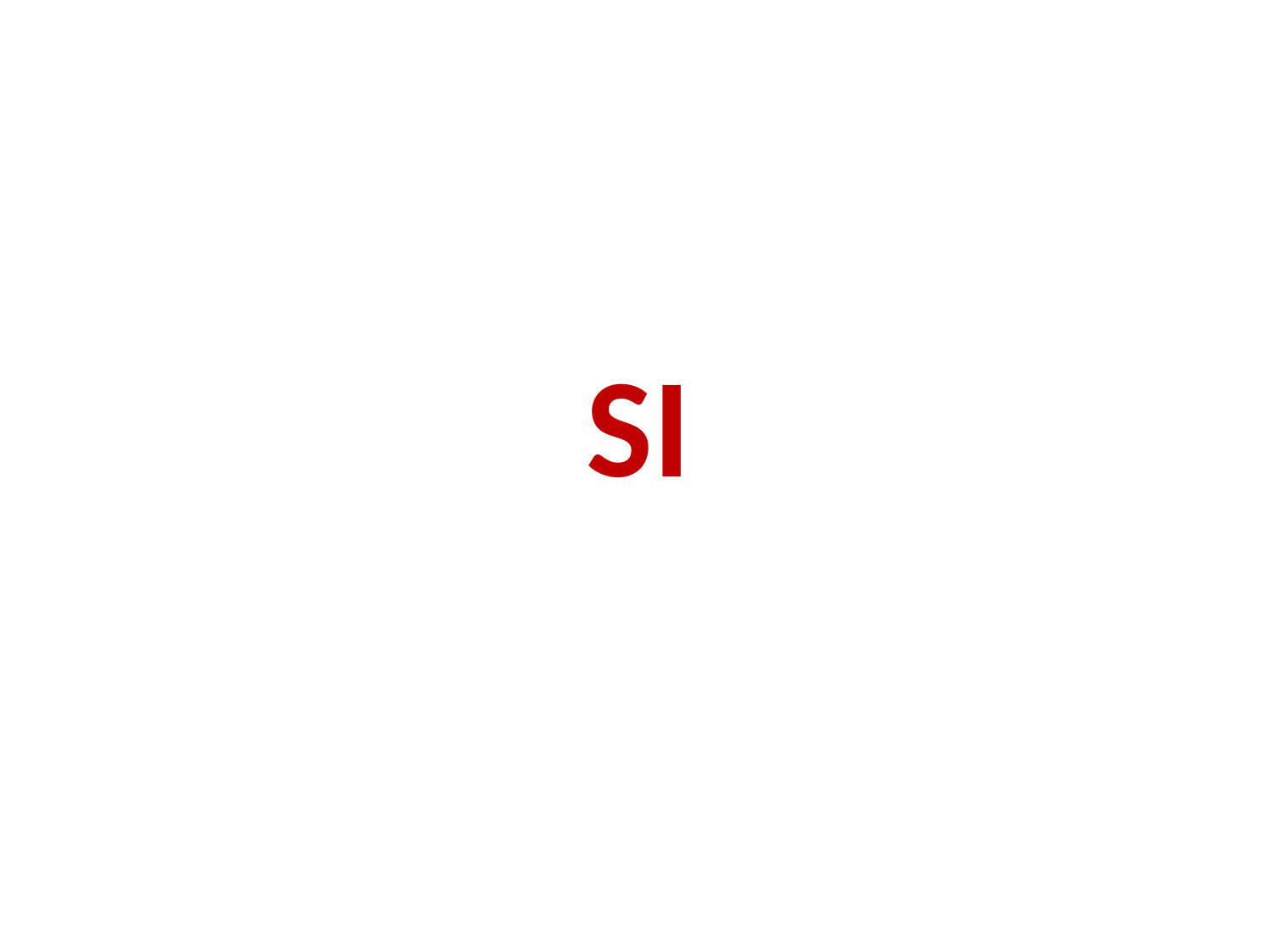

SI

## Slide 2
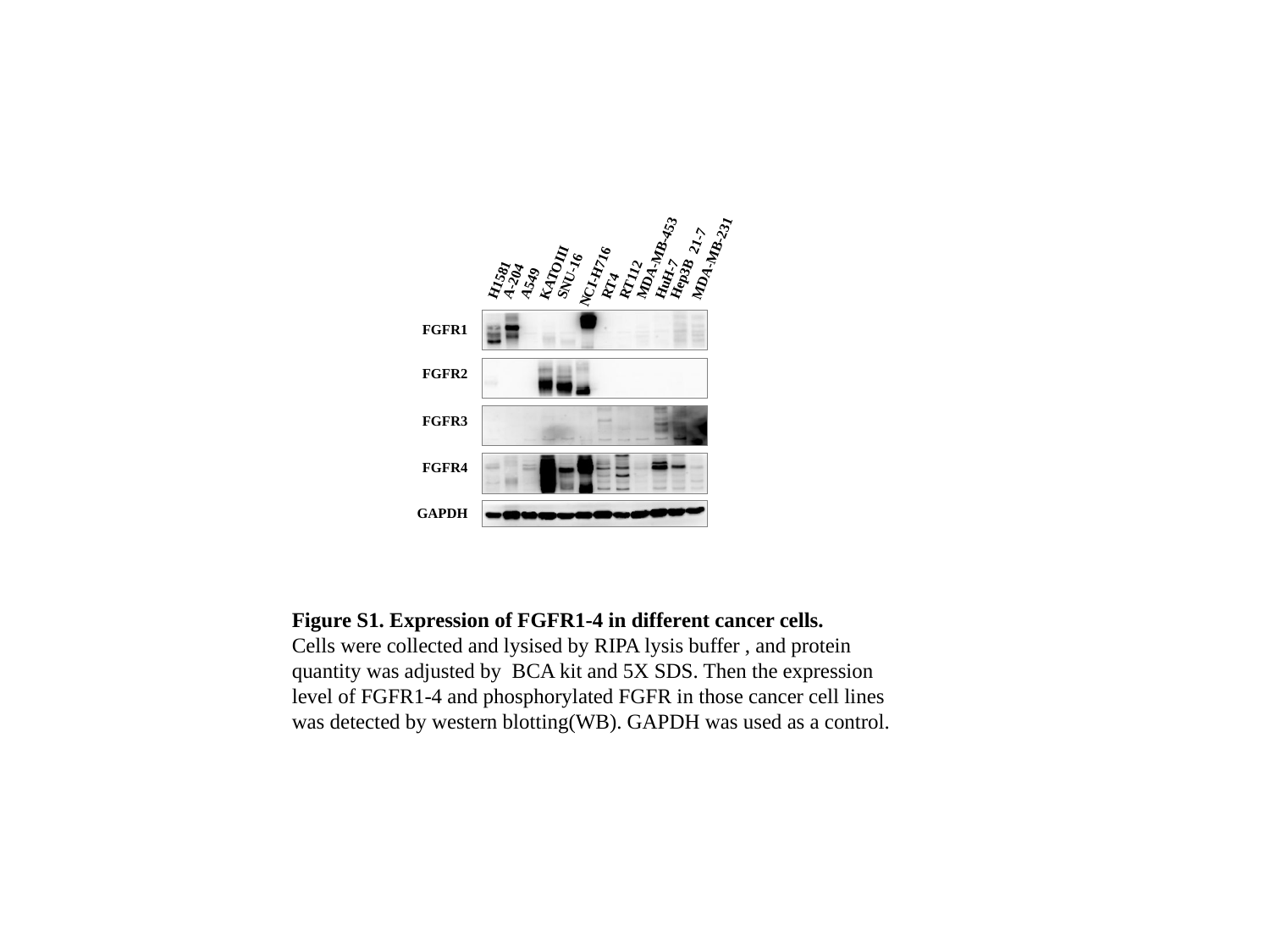

MDA-MB-453
MDA-MB-231
Hep3B 21-7
KATOIII
SNU-16
NCI-H716
A-204
A549
RT4
H1581
RT112
HuH-7
FGFR1
FGFR2
FGFR3
FGFR4
GAPDH
Figure S1. Expression of FGFR1-4 in different cancer cells.
Cells were collected and lysised by RIPA lysis buffer , and protein quantity was adjusted by BCA kit and 5X SDS. Then the expression level of FGFR1-4 and phosphorylated FGFR in those cancer cell lines was detected by western blotting(WB). GAPDH was used as a control.

## Slide 3
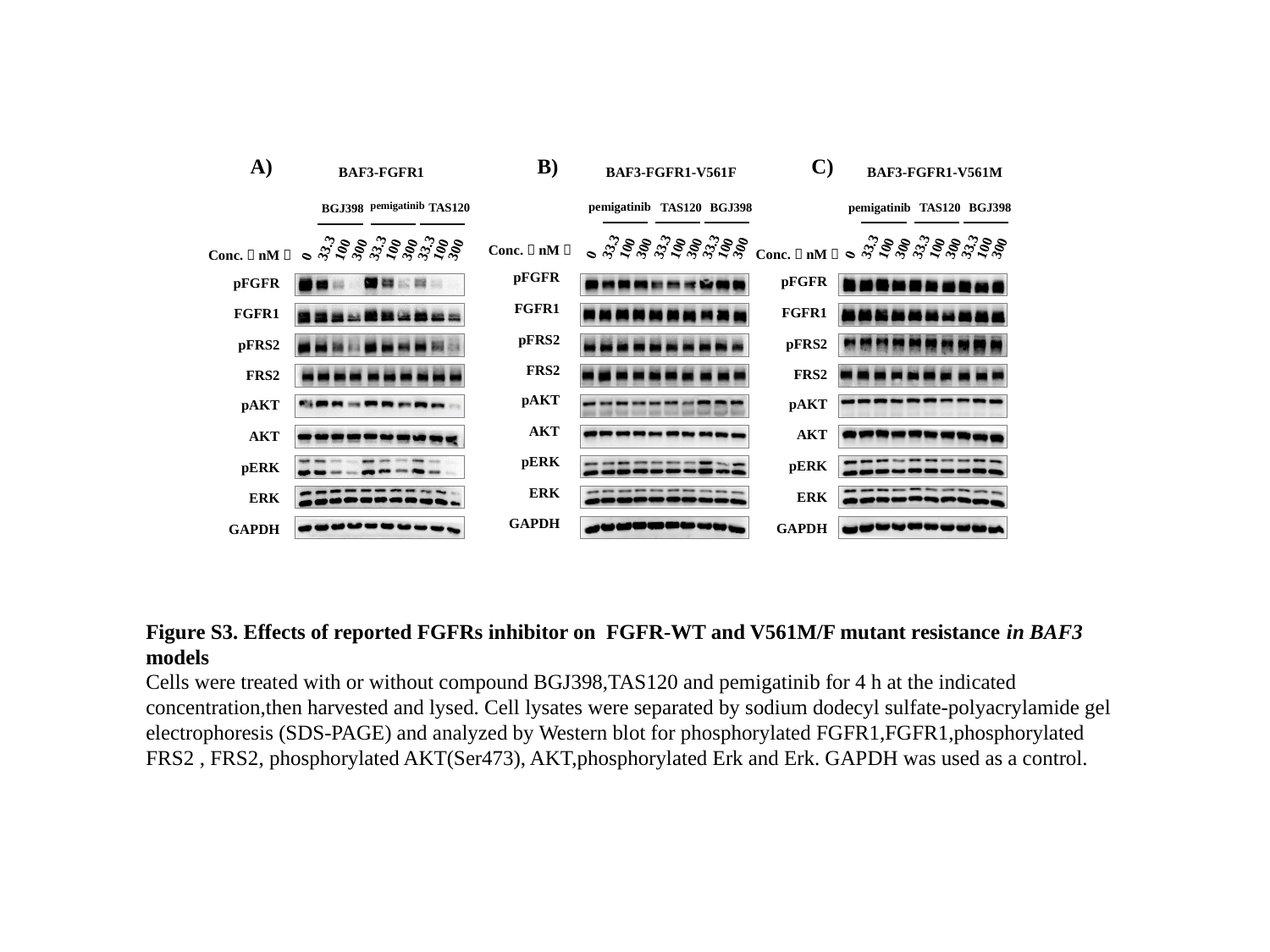

A)
B)
C)
BAF3-FGFR1
BAF3-FGFR1-V561F
BAF3-FGFR1-V561M
pemigatinib
TAS120
BGJ398
300
300
300
33.3
33.3
33.3
100
100
100
0
pemigatinib
BGJ398
TAS120
300
300
300
33.3
33.3
33.3
100
100
100
0
pemigatinib
BGJ398
TAS120
300
300
300
33.3
33.3
33.3
100
100
100
0
Conc.（nM）
Conc.（nM）
Conc.（nM）
pFGFR
pFGFR
pFGFR
FGFR1
FGFR1
FGFR1
pFRS2
pFRS2
pFRS2
FRS2
FRS2
FRS2
pAKT
pAKT
pAKT
AKT
AKT
AKT
pERK
pERK
pERK
ERK
ERK
ERK
GAPDH
GAPDH
GAPDH
Figure S3. Effects of reported FGFRs inhibitor on FGFR-WT and V561M/F mutant resistance in BAF3 models
Cells were treated with or without compound BGJ398,TAS120 and pemigatinib for 4 h at the indicated concentration,then harvested and lysed. Cell lysates were separated by sodium dodecyl sulfate-polyacrylamide gel electrophoresis (SDS-PAGE) and analyzed by Western blot for phosphorylated FGFR1,FGFR1,phosphorylated FRS2 , FRS2, phosphorylated AKT(Ser473), AKT,phosphorylated Erk and Erk. GAPDH was used as a control.

## Slide 4
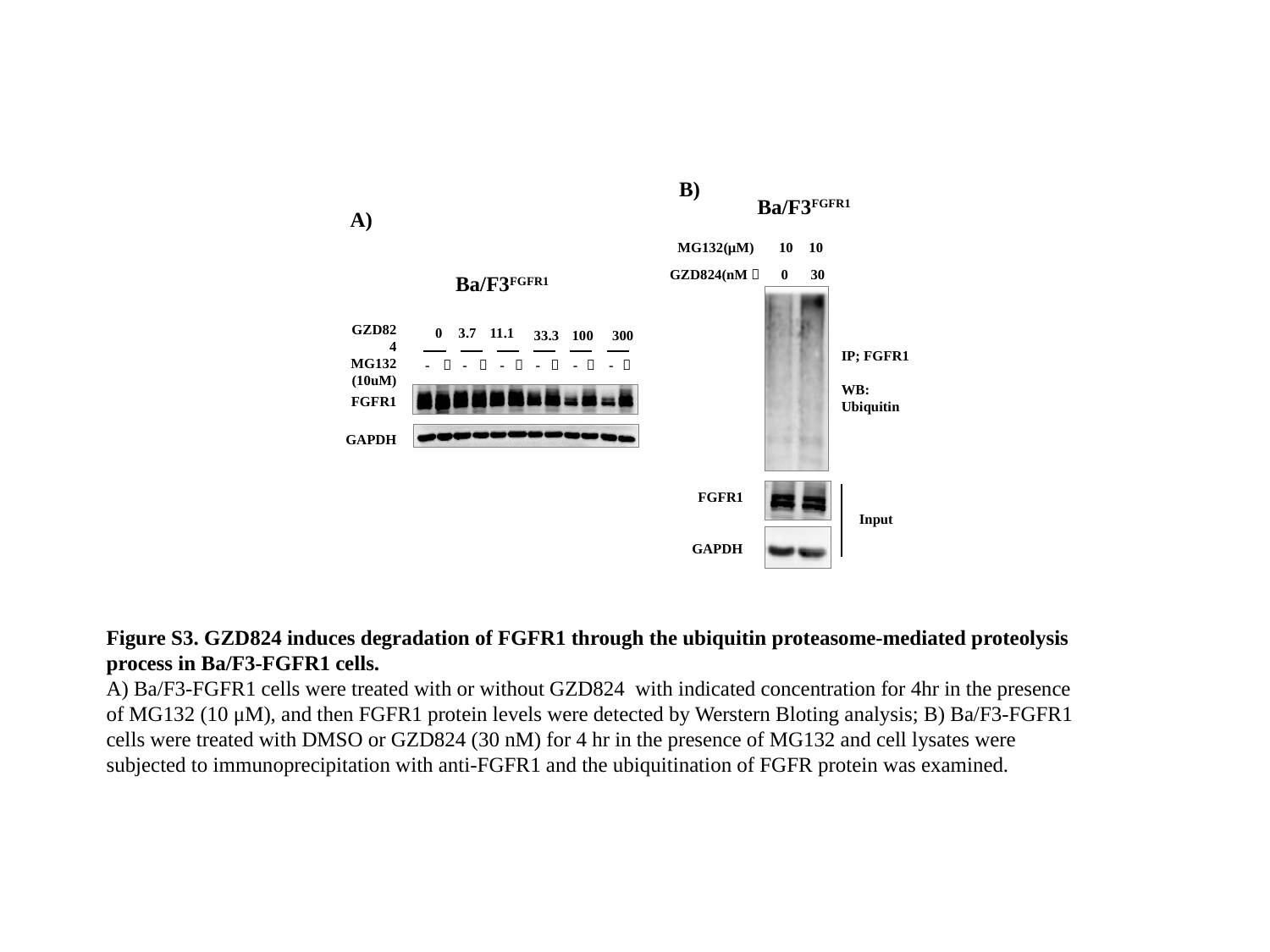

B)
Ba/F3FGFR1
A)
MG132(μM)
10
10
GZD824(nM）
0
30
Ba/F3FGFR1
GZD824
0
3.7
11.1
33.3
100
300
IP; FGFR1
WB: Ubiquitin
MG132 (10uM)
-
＋
-
＋
-
＋
-
＋
-
＋
-
＋
FGFR1
GAPDH
FGFR1
Input
GAPDH
Figure S3. GZD824 induces degradation of FGFR1 through the ubiquitin proteasome-mediated proteolysis process in Ba/F3-FGFR1 cells.
A) Ba/F3-FGFR1 cells were treated with or without GZD824 with indicated concentration for 4hr in the presence of MG132 (10 μM), and then FGFR1 protein levels were detected by Werstern Bloting analysis; B) Ba/F3-FGFR1 cells were treated with DMSO or GZD824 (30 nM) for 4 hr in the presence of MG132 and cell lysates were subjected to immunoprecipitation with anti-FGFR1 and the ubiquitination of FGFR protein was examined.

## Slide 5
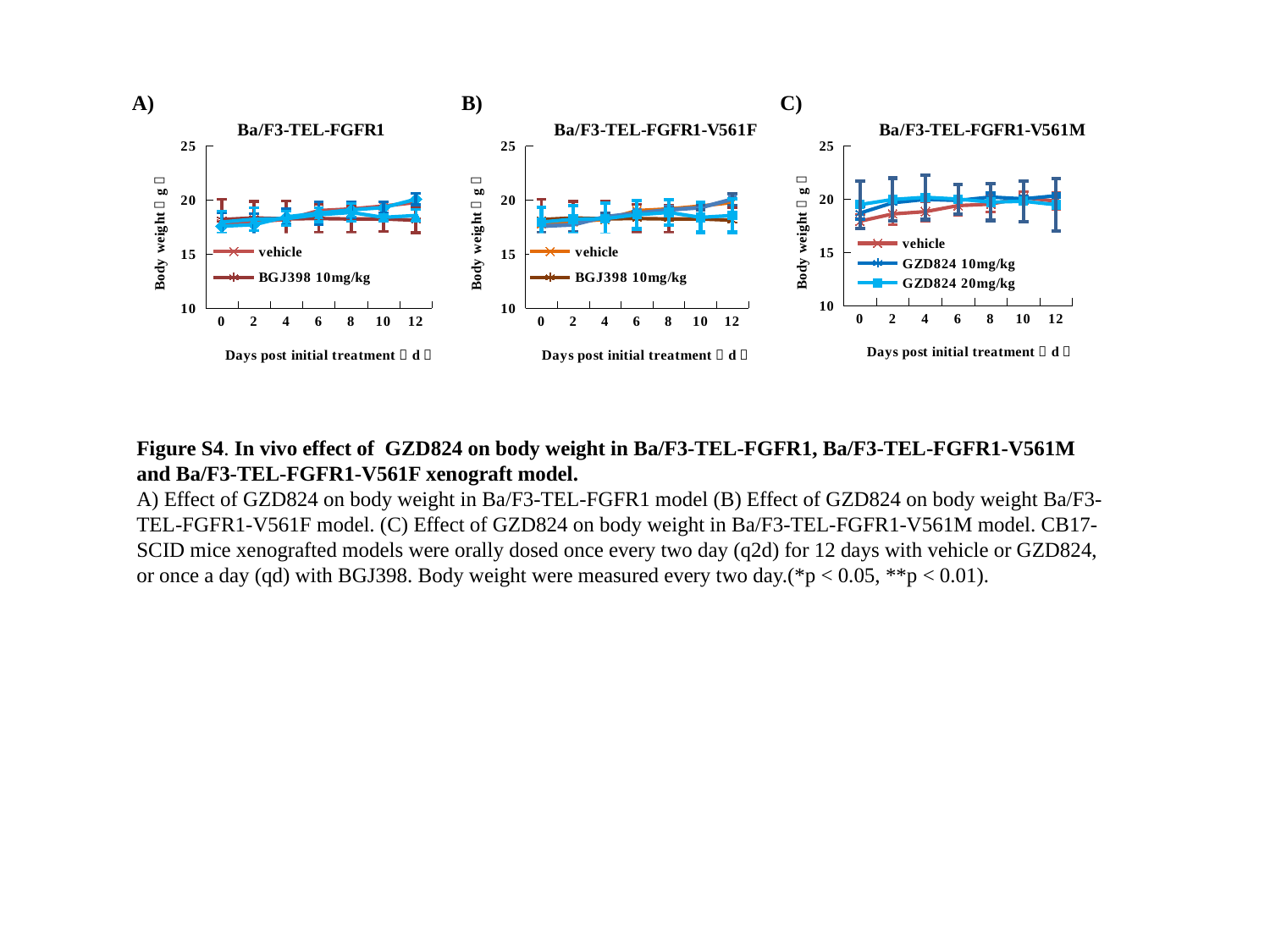

A)
B)
C)
### Chart: Ba/F3-TEL-FGFR1
| Category | vehicle | BGJ398 10mg/kg | GZD824 10mg/kg | GZD824 20mg/kg |
|---|---|---|---|---|
| 0 | 17.831666666666667 | 18.210000000000004 | 17.595 | 17.975 |
| 2 | 18.013333333333332 | 18.358333333333334 | 17.735 | 18.235 |
| 4 | 18.213333333333335 | 18.279999999999998 | 18.46 | 18.295 |
| 6 | 19.038333333333334 | 18.304999999999996 | 18.805 | 18.655 |
| 8 | 19.19 | 18.264999999999997 | 19.105 | 18.869999999999997 |
| 10 | 19.460000000000004 | 18.27 | 19.314999999999998 | 18.41 |
| 12 | 19.788333333333338 | 18.144 | 20.085 | 18.585 |
### Chart: Ba/F3-TEL-FGFR1-V561F
| Category | vehicle | BGJ398 10mg/kg | GZD824 10mg/kg | GZD824 20mg/kg |
|---|---|---|---|---|
| 0 | 17.831666666666667 | 18.210000000000004 | 17.595 | 17.975 |
| 2 | 18.013333333333332 | 18.358333333333334 | 17.735 | 18.235 |
| 4 | 18.213333333333335 | 18.279999999999998 | 18.46 | 18.295 |
| 6 | 19.038333333333334 | 18.304999999999996 | 18.805 | 18.655 |
| 8 | 19.19 | 18.264999999999997 | 19.105 | 18.869999999999997 |
| 10 | 19.460000000000004 | 18.27 | 19.314999999999998 | 18.41 |
| 12 | 19.788333333333338 | 18.144 | 20.085 | 18.585 |
### Chart: Ba/F3-TEL-FGFR1-V561M
| Category | vehicle | GZD824 10mg/kg | GZD824 20mg/kg |
|---|---|---|---|
| 0 | 17.901666666666667 | 18.659999999999997 | 19.455 |
| 2 | 18.57833333333333 | 19.630000000000003 | 19.935000000000002 |
| 4 | 18.796666666666667 | 19.945 | 20.145 |
| 6 | 19.365 | 19.845 | 19.97 |
| 8 | 19.5 | 20.165 | 19.689999999999998 |
| 10 | 20.12166666666667 | 19.994999999999997 | 19.77 |
| 12 | 19.776666666666667 | 20.285 | 19.445 |Figure S4. In vivo effect of GZD824 on body weight in Ba/F3-TEL-FGFR1, Ba/F3-TEL-FGFR1-V561M and Ba/F3-TEL-FGFR1-V561F xenograft model.
A) Effect of GZD824 on body weight in Ba/F3-TEL-FGFR1 model (B) Effect of GZD824 on body weight Ba/F3-TEL-FGFR1-V561F model. (C) Effect of GZD824 on body weight in Ba/F3-TEL-FGFR1-V561M model. CB17-SCID mice xenografted models were orally dosed once every two day (q2d) for 12 days with vehicle or GZD824, or once a day (qd) with BGJ398. Body weight were measured every two day.(*p < 0.05, **p < 0.01).

## Slide 6
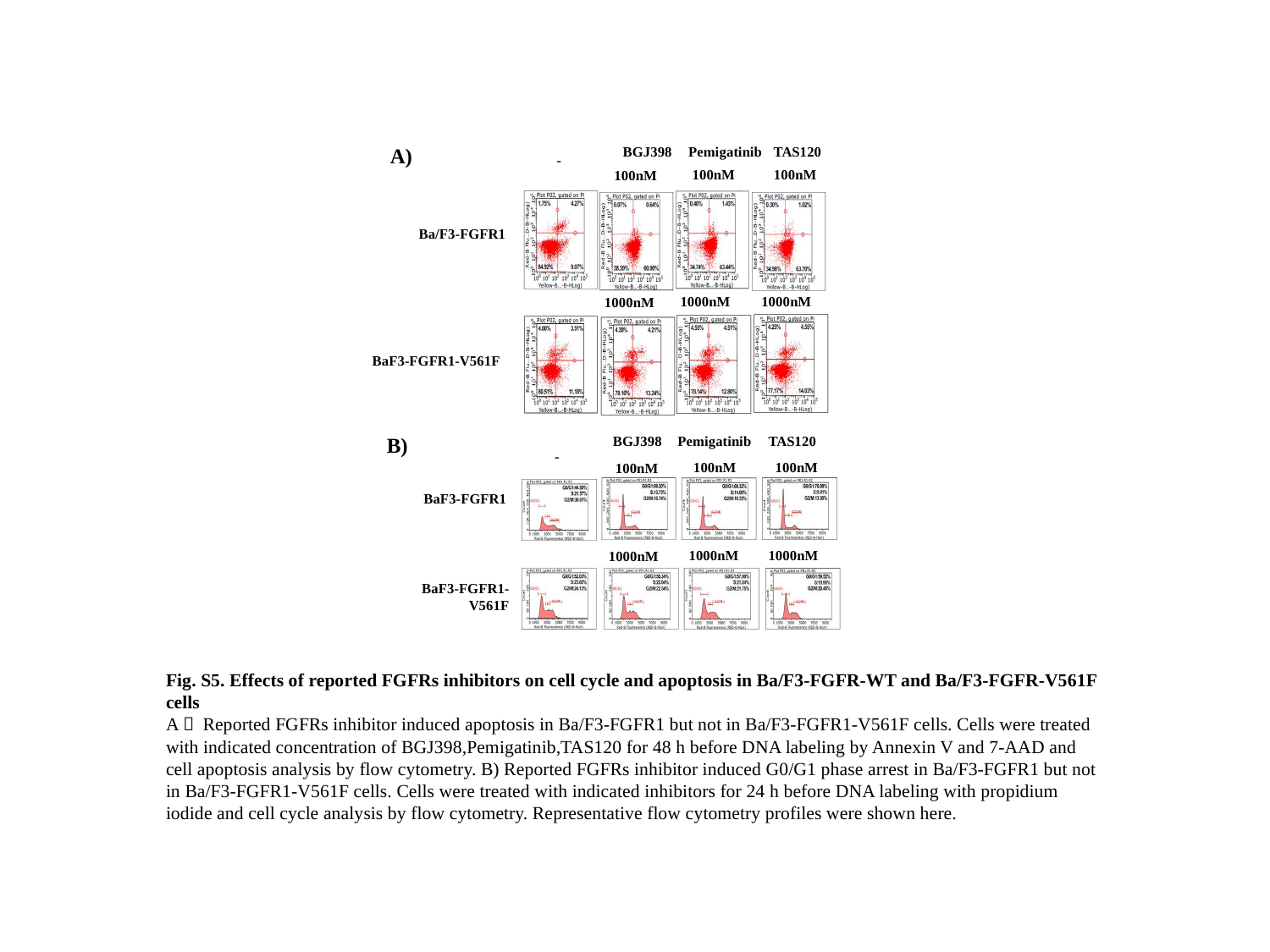

A)
BGJ398
Pemigatinib
TAS120
-
100nM
100nM
100nM
Ba/F3-FGFR1
1000nM
1000nM
1000nM
BaF3-FGFR1-V561F
B)
BGJ398
TAS120
Pemigatinib
-
100nM
100nM
100nM
BaF3-FGFR1
1000nM
1000nM
1000nM
BaF3-FGFR1-V561F
Fig. S5. Effects of reported FGFRs inhibitors on cell cycle and apoptosis in Ba/F3-FGFR-WT and Ba/F3-FGFR-V561F cells
A） Reported FGFRs inhibitor induced apoptosis in Ba/F3-FGFR1 but not in Ba/F3-FGFR1-V561F cells. Cells were treated with indicated concentration of BGJ398,Pemigatinib,TAS120 for 48 h before DNA labeling by Annexin V and 7-AAD and cell apoptosis analysis by flow cytometry. B) Reported FGFRs inhibitor induced G0/G1 phase arrest in Ba/F3-FGFR1 but not in Ba/F3-FGFR1-V561F cells. Cells were treated with indicated inhibitors for 24 h before DNA labeling with propidium iodide and cell cycle analysis by flow cytometry. Representative flow cytometry profiles were shown here.
